# Adverse drug event detection using natural language processing: A scoping review of supervised learning methods

**DOI:** 10.1371/journal.pone.0279842

**Published:** 2023-01-03

**Authors:** Rachel M. Murphy, Joanna E. Klopotowska, Nicolette F. de Keizer, Kitty J. Jager, Jan Hendrik Leopold, Dave A. Dongelmans, Ameen Abu-Hanna, Martijn C. Schut

**Affiliations:** 1 Department of Medical Informatics, Amsterdam UMC (location AMC), Amsterdam, The Netherlands; 2 Amsterdam Public Health Research Institute, Amsterdam, The Netherlands; 3 Department of Intensive Care Medicine, Amsterdam UMC (location AMC), Amsterdam, The Netherlands; National University of Science and Technology, PAKISTAN

## Abstract

To reduce adverse drug events (ADEs), hospitals need a system to support them in monitoring ADE occurrence routinely, rapidly, and at scale. Natural language processing (NLP), a computerized approach to analyze text data, has shown promising results for the purpose of ADE detection in the context of pharmacovigilance. However, a detailed qualitative assessment and critical appraisal of NLP methods for ADE detection in the context of ADE monitoring in hospitals is lacking. Therefore, we have conducted a scoping review to close this knowledge gap, and to provide directions for future research and practice. We included articles where NLP was applied to detect ADEs in clinical narratives within electronic health records of inpatients. Quantitative and qualitative data items relating to NLP methods were extracted and critically appraised. Out of 1,065 articles screened for eligibility, 29 articles met the inclusion criteria. Most frequent tasks included named entity recognition (n = 17; 58.6%) and relation extraction/classification (n = 15; 51.7%). Clinical involvement was reported in nine studies (31%). Multiple NLP modelling approaches seem suitable, with Long Short Term Memory and Conditional Random Field methods most commonly used. Although reported overall performance of the systems was high, it provides an inflated impression given a steep drop in performance when predicting the ADE entity or ADE relation class. When annotating corpora, treating an ADE as a relation between a drug and non-drug entity seems the best practice. Future research should focus on semi-automated methods to reduce the manual annotation effort, and examine implementation of the NLP methods in practice.

## 1. Introduction

Adverse drug events (ADEs) represent a significant clinical problem in healthcare, owing to the increasing multimorbidity and complexity of medical treatement. Therefore, improving medication safety has been set as a global patient safety challenge, with a goal to reduce the level of severe, avoidable harm related to medication by 50% over 5 years [1]. Since the pooled prevalence of ADEs in the hospital setting is twice as hight as the pooled prevalence in primary care (19% versus 8%) [2, 3], we focus on this more vulnerable patient population. In order to improve medication safety in hospitalized patients, hospitals need to have accurate and continuous insight into what type of ADEs occur in their inpatients including which subpopulations are at high ADE risk. Such information is crucial in order to gain better understanding of the medication, patients, and clinical processes that are most amenable to medication safety interventions and on which of these to focus their efforts.

One of the major barriers for gaining such insight is lack of a monitoring system that can routinely, rapidly and at scale detect ADEs in hospitalized patients [4]. Such a system would help to obtain information about ADEs that have occurred in hospitalized patients. Subsequently, this information could be used to predict ADE occurrence in future inpatients, supporting clinicians in timely ADE recognition. At present, most hospitals rely on voluntary reporting of ADEs by healthcare staff, yet numerous studies have shown that this approach detects less than 1% of all ADEs [5]. The more comprehensive ADE identification method—patient chart review by pharmacists–can identify up to 20 times more ADE but is prohibitively expensive and time-consuming [6, 7].

The widespread adoption of electronic health record (EHR) systems has led to repositories of digital patient data, creating the potential to use information technology to generate computerized ADE monitoring systems in hospitals for routine, rapid and continuous analysis of the vast amounts of data [8]. However, since most information about ADEs tend to be registered in EHRs as free text mentions in clinical narratives (such as progress notes or discharge letters), extensive processing and formatting of this data is needed in order for a computer to accurately analyse it [9, 10]. The use of natural language processing (NLP) may help to address these challenges.

NLP is a domain of computer science that uses computers to manipulate free text data in the context of a specific task [11]. NLP has been investigated in the clinical domain for a range of tasks, from extracting information on medication dosage to classifying cancer staging from pathology reports [12]. Regarding the task of detecting ADEs, the majority of NLP efforts focus on pharmacovigilance [9, 11]. Two recent literature reviews, one systematic and one narrative, on this topic have provided a strategic overview of the progress that has been made with NLP on pharmacovigilance using EHR data, as well as the challenges pertaining to such a task [9, 11]. Challenges highlighted in these studies include limited data sharing between healthcare organizations and in detecting ADEs that arise from polypharmacy (drug-drug interactions) [9, 11]. In addition, a recent scoping review on key use cases for artificial intelligence to reduce the frequency of ADEs, promising NLP applications are presented [13]. However, these reviews lack a detailed description of the steps needed to apply NLP for ADE detection using EHR data in the context of ADE monitoring in hospitalized patients, including critical appraisal of NLP methods used.

Furthermore, most previous studies on ADE detection using NLP have investigated detection of separate clinical entities such as diagnoses, drug names and associated attributes such as dose, route, frequency, or looked at ADEs in the context of pharmacovigilance and post-market surveillance using predominantly spontaneous reporting databases. However, when detecting ADE mentions in clinical notes, both the drug and adverse event must be detected as well as the causality that links them. This causal element is missing when searching for separate entities. This complexity is often overlooked. In addition, spontaneous reporting databases include data which differ greatly from clinical narratives in EHR databases.

Therefore, we have conducted a scoping review to close this knowledge gap. Our aim is to examine the use of NLP methods in detecting ADE mentions in clinical notes in order to improve medication safety in hospitalized patients. We examine supervised learning methods since these are the most common type of machine learning applied in the medical domain. Focusing on the hospital setting enables a better comparison of the NLP methods presented in our scoping review. This work also includes a structured framework and critical appraisal of the included studies. The results give insight into strengths and limitations of current NLP applications for the task of ADE detection in hospitalized patients, and provide guidance on how to move forward to create NLP-based systems fit for purpose of monitoring medication safety in hospitals. Overall, this paper aims to serve as a reference point for both data scientists, clinicians and pharmacists as well as for decision-makers in the clinical medication safety domain, particularly from the methodological point of view.

## 2. Methods

### 2.1 Approach

**Fig 1:**
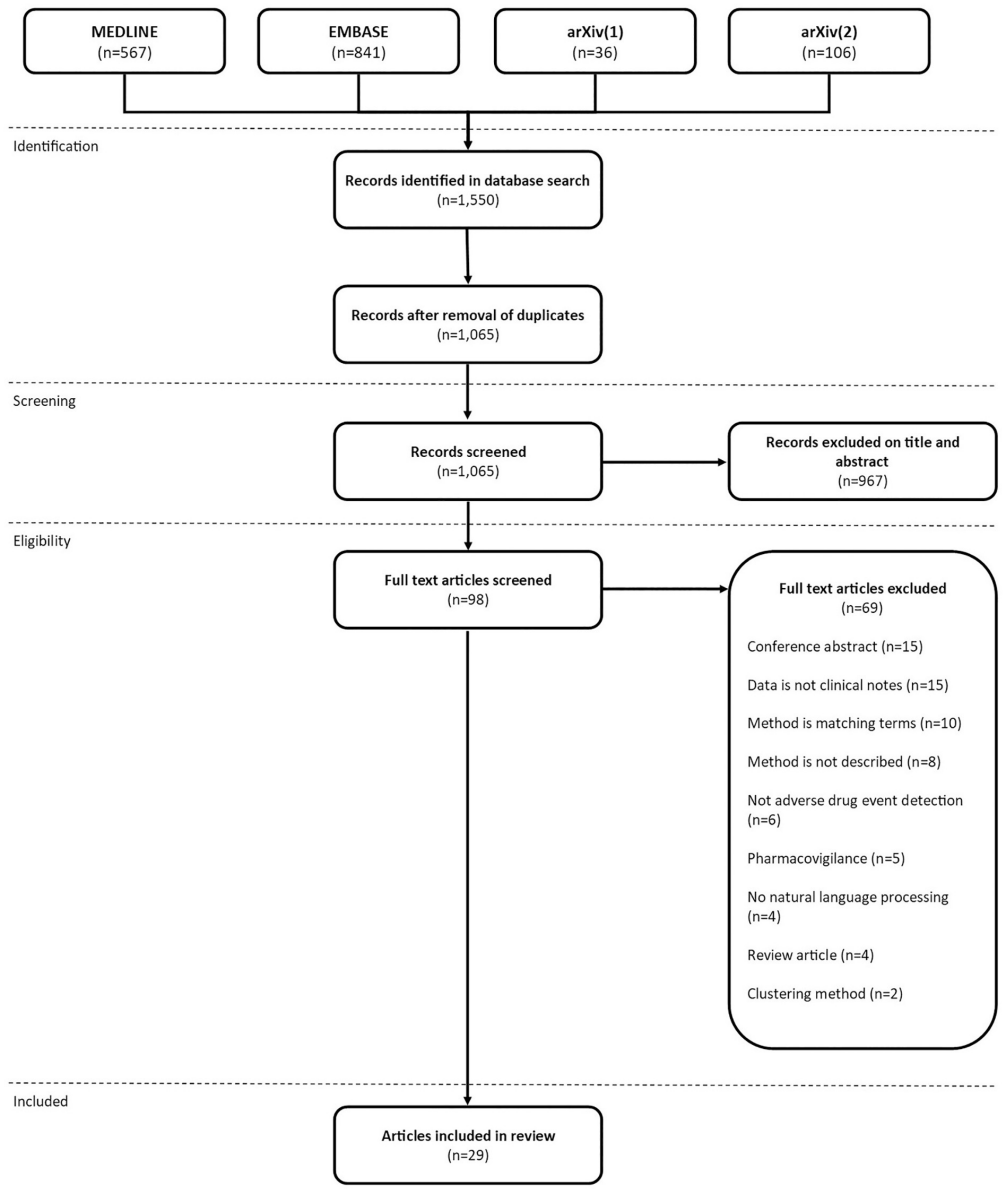
PRISMA flow diagram.

Our approach for conducting the scoping review is based on a set of recommendations outlined by Arksey and O’Malley [14] and the additional recommendations on this framework proposed by Levac et al. [15]. We further implemented recommendations specific to methodology scoping reviews, including identification of search terms, iterative search technique, and features to extract [16]. For reporting we have used the Preferred Reporting Items for Systematic reviews and Meta-Analyses extension for Scoping Reviews (PRISMA-ScR) checklist [17]. The checklist can be found in S1 Checklist. The corresponding PRISMA flow diagram is shown in Fig 1 and further explained in Section 3.1.

**Fig 2:**
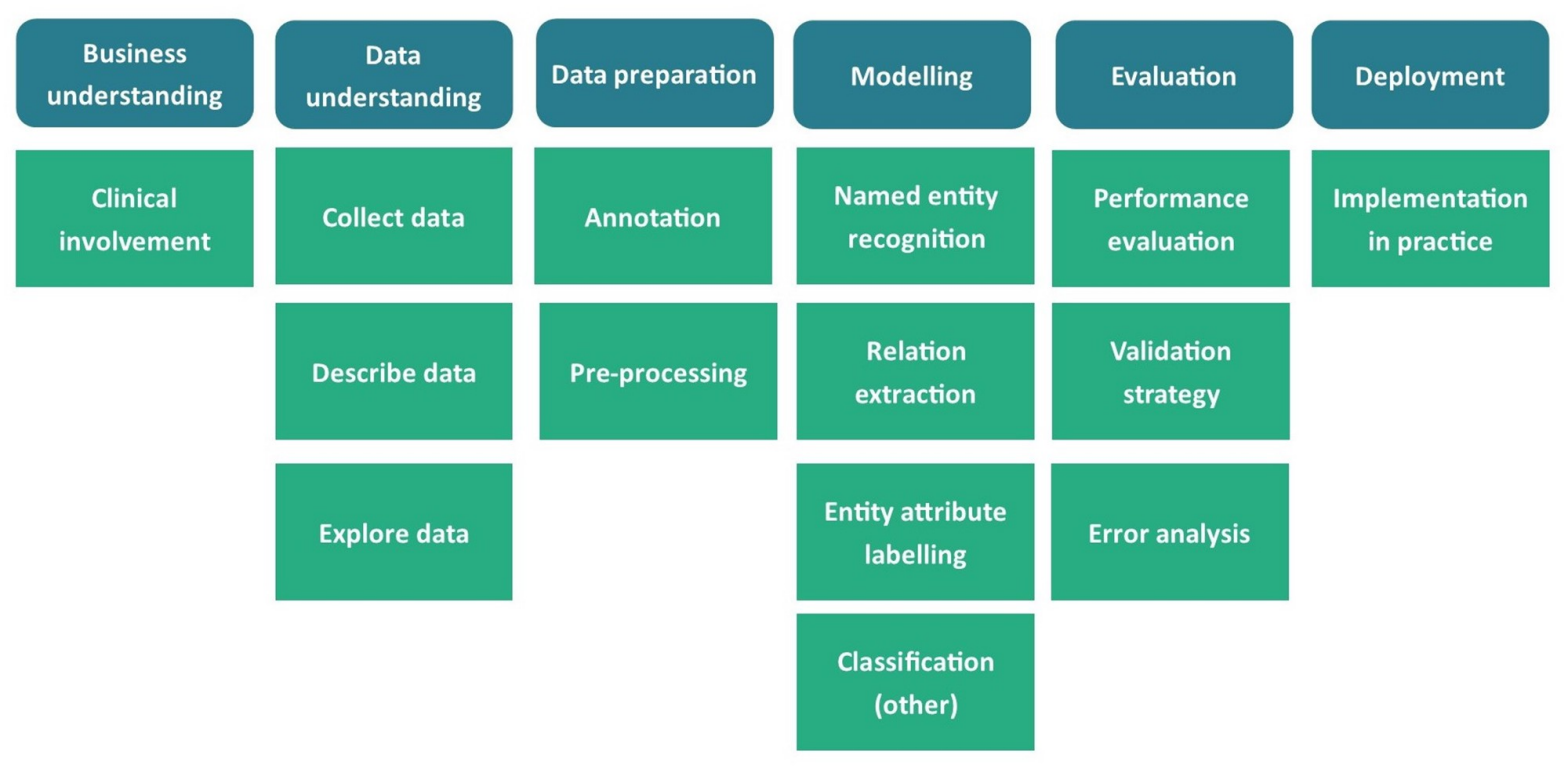
A framework for NLP workflow in clinical setting according to CRISP-DM reference model [18].

To create a framework for the review and critical appraisal of the included articles, we have used the Cross-industry standard process for data mining (CRISP-DM) [18] as a reference model to describe the stages and steps of the workflow to use NLP for ADE detection in hospitalized patients. This framework for NLP workflow is depicted in Fig 2. Box 1 provides a glossary of NLP terms used in this review.

**Box 1. Glossary of natural language processing and technical terms used in this review.**

**Table:** 

Term	Definition
*NLP terms and tasks*	
**Supervised machine learning**	Task of learning a model from labeled training data consisting of a set of training examples.
**Unsupervised machine learning**	Task of learning from data that does not have any labels; therefore it seeks patterns that naturally occur in the dataset.
**Corpus**	A collection of texts forming a dataset.
**Text pre-processing**	A range of techniques designed to clean and format the data so that it can be further analyzed, for example, sentence boundary detection, tokenization, part-of-speech tagging. Examples of frequently used libraries and tools for these purposes include StanfordNLP [19], Natural Language ToolKit (nltk) [20], openNLP [21] and spaCy [22].
**Sentence boundary detection**	A technique to determine where one sentence ends and the next sentence begins; also known as sentence segmentation.
**Tokenization**	Breaking down text into units known as tokens; a token may be a word, part of word, or punctuation.
**Part-of-speech (POS) tagging**	Categorizing tokens in a text with their corresponding part of speech, such as noun, verb, adjective, et cetera.
**Annotation**	The process of applying predefined labels or categories to text data; can be performed at the level of documents, sentences, phrases, or words. At the document or sentence level, labels are typically binary, for example: presence or absence of an ADE mention in the text; at the word or phrase level assigned labels are commonly multi-category (see Entity and Relation).
**Classification**	Assigning a category to data; can be binary (positive/negative) or multi-class (for example, “drug”, “diagnosis”, “symptom”).
**Entity**	A term or phrase in the text representing information to be extracted; in the clinical context this often includes diagnoses (“acute myocardial infarction”), symptoms (“fever”), and drug names (“morphine”).
**Named entity recognition (NER)**	A task of identifying and classifying entities in the text using a model. Examples of frequently used tools for NER include StanfordNLP [19], Natural Language ToolKit (nltk) [20], openNLP [21] and spaCy [22].
**Named entity normalization (NEN)**	Also called medical concept normalization, this is the task of matching entities to concepts in a medical terminology such as SNOMED-CT or ICD-10.
**Entity attribute**	A characteristic which describes an entity; for example, negation (“*denies* chest pain”), speculation (“*possible* lung neoplasm”), laterality (“*right* eye”).
**Entity attribute labelling**	A task of identifying and classifying attributes of entities in the text using a model; sometimes called concept attribute labelling.
**Relation**	A relationship that exists between two entities; for example, between a symptom entity and a drug entity possible relations include ADE and Indication.
**Relation extraction**	Identifying and classifying relationships between named entities using a model.
*Performance measures and dataset characteristics*	
**True positives**	A true positive (TP) is an instance that is correctly classified as positive; for example, the correct identification of an ADE in a text.
**False positives**	A false positive (FP) is an instance that is incorrectly classified as positive; for example, the identification of an ADE in a text where the text does not mention an ADE.
**True negatives**	A true negative (TN) is an instance that is correctly classified as negative; for example, the correct identification that a text does not mention an ADE.
**False negatives**	A false negative (FN) is an instance that is incorrectly classified as negative; for example, the incorrect identification that a text does not mention an ADE when the text does refer to an ADE.
**Confusion matrix**	A table of TP, FP, TN, and FN that is used to cross-tabulate the true and predicted classes that is used in machine learning to show the performance of a classification model.
**Accuracy**	Also called classification accuracy.
	$\frac{TP+TN}{TP+FN+TN+FP}$
**Precision**	Also called Positive Predictive Value (PPV).
	$\frac{TP}{TP+FP}$
**Recall**	Recall minimizes the impact of False Negatives and is a good metric for imbalanced data because it focuses on the minority class. Also referred to as sensitivity.
	$\frac{TP}{TP+FN}$
**F1 score**	The harmonic mean of precision and recall. The harmonic mean uses the reciprocals of the values and therefore minimizes the impact of large outliers; therefore, in order to have a high F1 score, you need to have both a high precision and a high recall.
**ROC curve**	This plot illustrates how well a binary classifier performs as its discrimination threshold is varied between 0 and 1. It compares the true positive rate and the false positive rate as the threshold changes. The area under this curve is often used in machine learning to quantify the discriminative ability of a model and to compare models.
*Other terms*	
**Imbalanced data**	A dataset with data belonging to distinct classes, where the size of one class is much larger than the other class (the precise difference between the groups in order to qualify as imbalanced data is not explicitly defined, but often data with a ratio of 1 (or less) to 10 is considered imbalanced). Imbalance data are common in ADE research.
**Internal validation**	Quantifies the performance of a model on unseen data from the same population as the training data.
**External validation**	Demonstrates how well a trained model performs on an external dataset. Important because it shows how well a model generalizes to new data.
**Overfitting and underfitting**	Overfitting is where a model learns the training data too well, which negatively impacts its performance on new data and means it cannot generalize well. In contrast, underfitting is where a model does not sufficiently learn from the training data. An underfit model performs poorly on the training data and also cannot generalize well to new data. Ideally the aim is to select a model that neither under- nor overfits to the training data.
**Shared task challenge**	A competition in which a common dataset is provided to participants with the goal of applying a technique to achieve a specific task, usually with the aim to increase engagement with the problem. For example: 2018 National NLP Clinical Challenges (n2c2) [23], and First Natural Language Processing Challenge for Extracting Medication, Indication, and Adverse Drug Events from Electronic Health Record Notes (MADE 1.0) [24].

### 2.2 Information sources

We used two types of information sources. The first type of source was peer-reviewed literature databases (MEDLINE and EMBASE). The search in these databases was most recently conducted on 1^st^ July 2021.

The second type of source was an open access archive and pre-print server for scholarly articles in the fields of computer science, statistics, and quantitative biology (among others), named arXiv. Articles submitted to arXiv are not peer-reviewed. This source was included to look for state-of-the-art technical literature. This search was most recently conducted on 1^st^ July 2021.

### 2.3 Search strategy

The search strategy was centred on three key themes, namely natural language processing, clinical narratives, and adverse drug events. Our choice of search terms was made following testing of individual search terms and consulting other reviews in NLP of clinical narratives [12, 25]. We did not employ any date or other types of filters to the search queries. The full search strategy is available in S1 File.

### 2.4 Selection of sources of evidence

The initial search results were sequentially de-duplicated in EndNote [26]. The title and abstract screening was performed using Rayyan, a web-based tool that facilitates collaboration in the screening process [27]. The screening was conducted by RMM with MCS reviewing 20% of the decisions to ensure that concordance was achieved. MCS was not blinded to the decisions made by RMM. Where decisions differed or were uncertain, JEK made the final decision.

The full text of the remaining articles was then reviewed to identify those that matched the inclusion and exclusion criteria. The full text review was performed by RMM, with a 10% sample blind reviewed by JHL. Where decisions differed or were uncertain, JEK made the final decision.

### 2.5 Eligibility criteria

To keep the information retrieved relevant and specific to our aim of detecting ADE mentions in clinical notes in order to improve medication safety in hospitalized patients, we chose to exclude articles that use clustering methods to find patterns of ADEs in clinical notes–this is commonly done with unsupervised learning. The output of such methods is primarily groups of patients with a distinguishing adverse event. Such output does not align with our aim. Similarly, we also chose to exclude articles that used data from the primary care setting and data from pharmacovigilance sources, as this data differs in several ways from inpatient clinical notes. Pharmacovigilance data, such as spontaneous reporting system data or drug labels, tend to have more formal and structured language, and the data is documented for a different purpose (signal detection or information dissemination rather than communicating with colleagues). The spontaneous reporting system data can be semi-structured (i.e. the drug name may be selected from a predefined list), unlike clinical notes where reference to the drug could be given by the brand name, drug name, drug group, or simply an abbreviation (e.g. “AKI due to AB”, meaning “acute kidney injury due to antibiotics”). Notably, the purpose is to report an ADE; therefore each spontaneous report should contain an ADE mention. In contrast, most inpatient clinical notes will not contain an ADE mention. Primary care and hospital care narratives differ greatly in terms of their frequency, structure, style, and language used, which merits a separate review of NLP methods applied to primary care and hospital care narratives. While these other types of data can contribute to the overall performance of an NLP pipeline for ADE detection, we chose to focus on the articles that use only clinical narratives from EHRs, to align closely with our aim.

We applied the following eligibility criteria to our search results.

1. Articles that describe NLP application for ADE detection in clinical narratives in EHRs of hospitalized patients were included.
2. Articles that used a list of terms to search for ADEs in clinical narratives were excluded.
3. Articles with NLP where the underlying method was not described were excluded.
4. Articles describing clustering methods on unlabelled data were excluded.
5. Conference papers were included, but conference abstracts that were insufficiently detailed were excluded.
6. Articles that used literature databases, drug labels, or spontaneous reporting systems as the ADE data source were excluded.
7. Articles that used primary care or community care narratives were excluded.
8. Articles that combined clinical narratives with spontaneous reporting systems to detect signals in pharmacovigilance were excluded.

### 2.6 Data charting and critical appraisal process

The data extraction form was based on relevant items from the CHecklist for critical Appraisal and data extraction for systematic Reviews of prediction Modelling Studies (CHARMS) and Prediction model Risk Of Bias Assessment Tool (PROBAST) reporting guidelines [28, 29]. In addition we included items recommended by Kersloot et al. [30] for the evaluation and validation of NLP algorithms.

Feedback from MCS and JEK on a sample extraction was used to produce the final data extraction chart. RMM completed the data charting and critical appraisal. The data was charted in Microsoft Excel and summarized using Microsoft Excel and RStudio.

### 2.7 Data items

The full list of items for which we sought to extract data is shown in Table 1. When extracting data relating to clinical involvement, we looked for any explicit mention in the manuscript of a clinical role (physician, pharmacist, nurse, medical student, or allied health professional) contributing in any way to the study.

**Table 1:** List of items extracted.

Topic	Item
**Article characteristics**	Year of publication
	Country of origin
	Author affiliations
	Journal or publication
**Business understanding**	Clinical involvement
**Data understanding**	Origin of data
	Language of data
	Type of data
	Quantity of data
**Data preparation**	Annotation
	Pre-processing
**Modelling**	Named entity recognition
	Relation extraction
	Entity attribute labelling
	Classification (other)
**Evaluation**	Performance evaluation measures
	Validation strategy
	Error analysis
**Deployment**	Implementation of the system in clinical practice

## 3. Results

### 3.1 Selection of sources of evidence

The final search yielded 1,550 articles. We removed 485 duplicates leaving 1,065 articles for title and abstract screening. We excluded 967 articles during title and abstract screening and 69 articles during full text screening, leaving 29 articles for data extraction. Fig 1 illustrates the selection process.

The main reason to exclude articles during the screening stages was that the data used in the article was not clinical narratives (n = 570). Other common reasons for exclusion were that the articles were not about ADEs (n = 243) or not about NLP (n = 77).

### 3.2 Characteristics of sources of evidence

**Fig 3:**
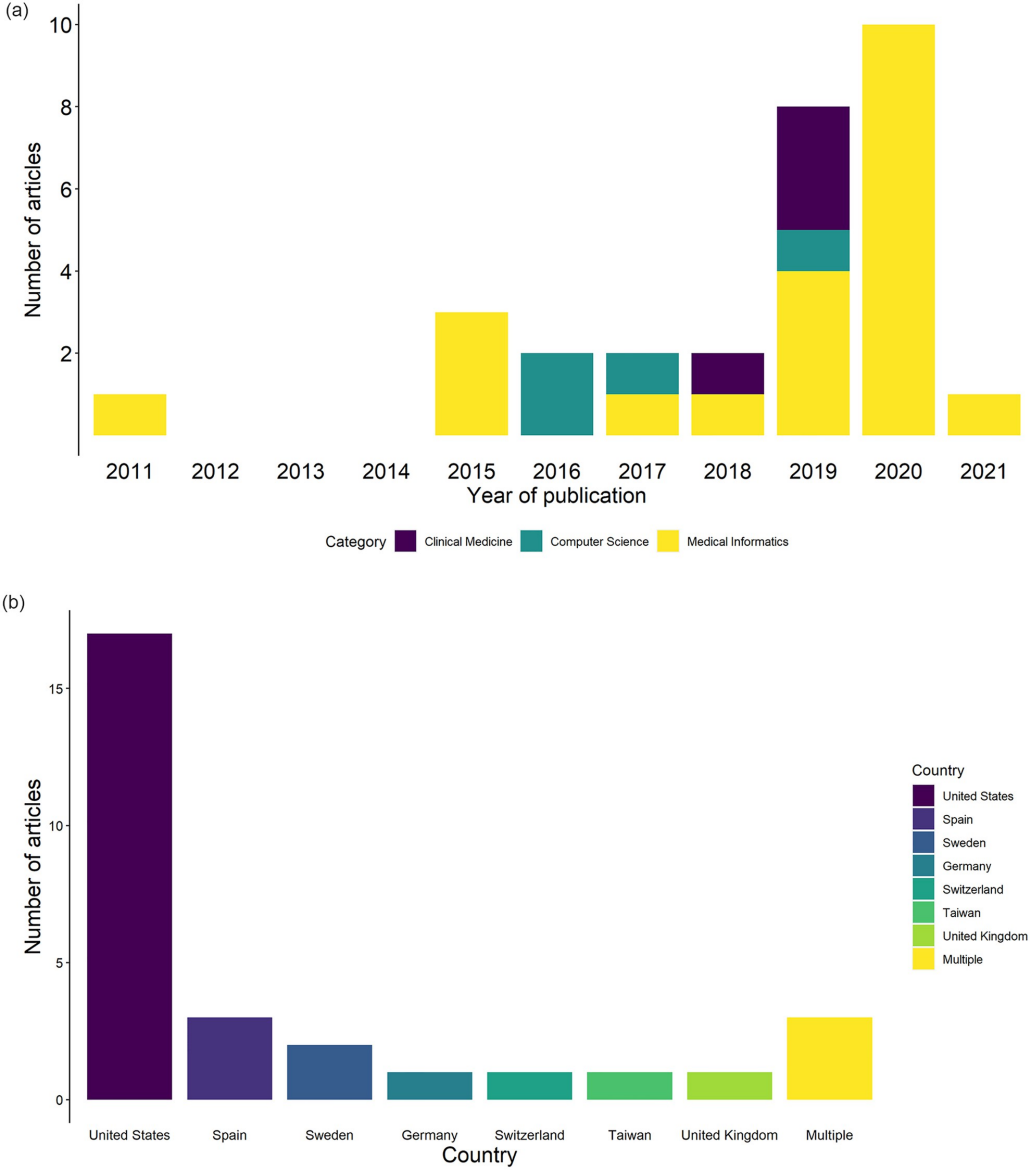
**a.** Number of articles published each year; search was conducted on 1^st^ July 2021. **b.** Number of articles by country of author institution.

The 29 included articles were published between 2011 and 2021 (see Fig 3a); note that we did not apply date filters to the searches. The journal category was identified as per the procedure outlined by Sheikhalishahi et al. [25] Studies were undertaken primarily in the United States (n = 17; 58.6%); three studies involved international collaboration (Fig 3b).

### 3.3 Summary of main findings

To summarize, Table 2 outlines the number of articles reporting items in the framework for NLP workflow (Fig 2) that we created to appraise the papers.

**Table 2:** Operational assessment of the included articles against the proposed framework.

Topic	Item	Number of articles	References
**Business understanding**	Clinical involvement	9 (31%)	[31–39]
**Data understanding**	Dataset description	29 (100%)	All included articles
**Data preparation**	Annotation	10 (34.5%)	[31–33, 36–42]
	Pre-processing	25 (86.2%)	[31, 33–39, 42–58]
**Modelling**	Named entity recognition	17 (58.6%)	[33, 34, 37, 41, 43–49, 53–58]
	Relation extraction	15 (51.7%)	[33–35, 38, 39, 43, 45, 49–53, 56, 58, 59]
	Entity attribute labelling	3 (10.3%)	[31, 33, 38]
	Classification (other)	5 (17.2%)	[31, 32, 36, 40, 42]
**Evaluation**	Performance evaluation measures	29 (100%)	All included articles
	Validation strategy–internal	18 (62.1%)	[32, 33, 35, 36, 39, 42–44, 46–49, 51, 52, 54, 57–59]
	Validation strategy–external	1 (3.4%)	[50]
	Error analysis	12 (41.4%)	[33, 34, 36, 37, 39, 41, 43, 45, 49, 50, 57, 58]
**Deployment**	Implementation in practice	0	

### 3.4 Business understanding

#### 3.4.1 Clinical involvement

Types of clinical consultation or involvement mentioned included annotation of notes, annotation scheme design, and clinical chart review.

### 3.5 Data description

Publicly available datasets were used in 15 (51.7%) of the studies, while 14 (48.3%) studies made use of data from their own institutions. The vast majority of datasets have a size of hundreds or thousands documents (see Table 3). Only one (2.6%) study used tens of thousands of clinical narratives, despite the fact that machine learning approaches are data hungry in the sense that their performance is strongly correlated with the amount of training data available [60]. The number of ADEs in the datasets (where reported) was in the hundreds (range 144–1,940, see Table 3).

**Table 3:** Dataset characteristics.

Definition of ADE in the dataset	Lead author	Data language	Dataset size in number of notes	Number of labelled documents	Number of ADEs
**Named entities**	Belousov, M. [57]	English	505	505	1,584
	Chapman, A.B. [58]	English	1,089	1,089	1,940
	Chen, L. [43]	English	505	505	1,579^a^
	Dai, H.J. [44]	English	505	505	1,568^a^
	Dandala, B. [45]	English	505	505	1,584
	Guan, H. [59]	English	1,092 [dataset 1]	1,092 [dataset 1]	*Not stated*
			505 [dataset 2]	505 [dataset 2]	
	Jagannatha, A.N. 2016a [46]	English	1,154	1,154	1,807
	Jagannatha, A.N. 2016b [47]	English	780	780	905
	Ju, M. [48]	English	505	505	959^b^
	Kim, Y. [34]	English	505	505	1,584
	Li, F. 2018 [49]	English	1,089	1,089	*Not stated*
	Li, F. 2019 [50]	English	1,089 [dataset 1]	1,089 [dataset 1]	*Not stated*
			485 [dataset 2]	485 [dataset 2]	
			1,243 [dataset 3]	1,243 [dataset 3]	
	Mitra, A. [37]	English	1,079	1,079	*Not stated*
	Munkhdalai, T. [41]	English	791	791	*Not stated*
	Wei, Q. [53]	English	505	505	*Not stated*
	Wunnava, S. [54]	English	1,089	1,089	*Not stated*
	Yang, X. 2020 [56]	English	505	505	1,584
	Yang, X. 2019 [55]	English	1,089	1,089	*Not stated*
**Relations between entities**	Henriksson, A. [33]	Swedish	3,690	400	144
	Oronoz, M. [39]	Spanish	75	75	228
	Santiso, S. 2019a [51]	Spanish	75	75	147
	Santiso, S. 2019b [35]	Spanish	75 [dataset 1]	75 [dataset 1]	110 [dataset 1] 338 [dataset 2]
			267 [dataset 2]	267 [dataset 2]	
	Sohn, S. [38]	English	237	237	335
	Taewijit, S. [52]	English	50,998	*Not stated*	*Not stated*
**Patient labelling**	Gupta, S. [36]	English	9,924	724^c^	335
	Rebane, J. [42]	Swedish	*Not stated*	*Not stated*	*Not stated*
**Document annotation**	Boyce, R.D. [31]	English	1,944	1,035	675
	Foufi, V. [40]	French	300	87	441
**Sentence annotation**	Gaebel, J. [32]	German	5	5	*Not stated*

^a^ These studies used the dataset from the n2c2 shared task challenge. According to the challenge organizers, there were 1,584 ADEs in the 505 notes: 959 in the training set and 625 in the test set [23]. The numbers reported by the authors do not seem to match with the numbers reported by the challenge organizers.

^b^ This study used the dataset from the n2c2 shared task challenge and reported only the number of ADEs in the training set.

^c^ Refers to number of patients.

**Fig 4:**
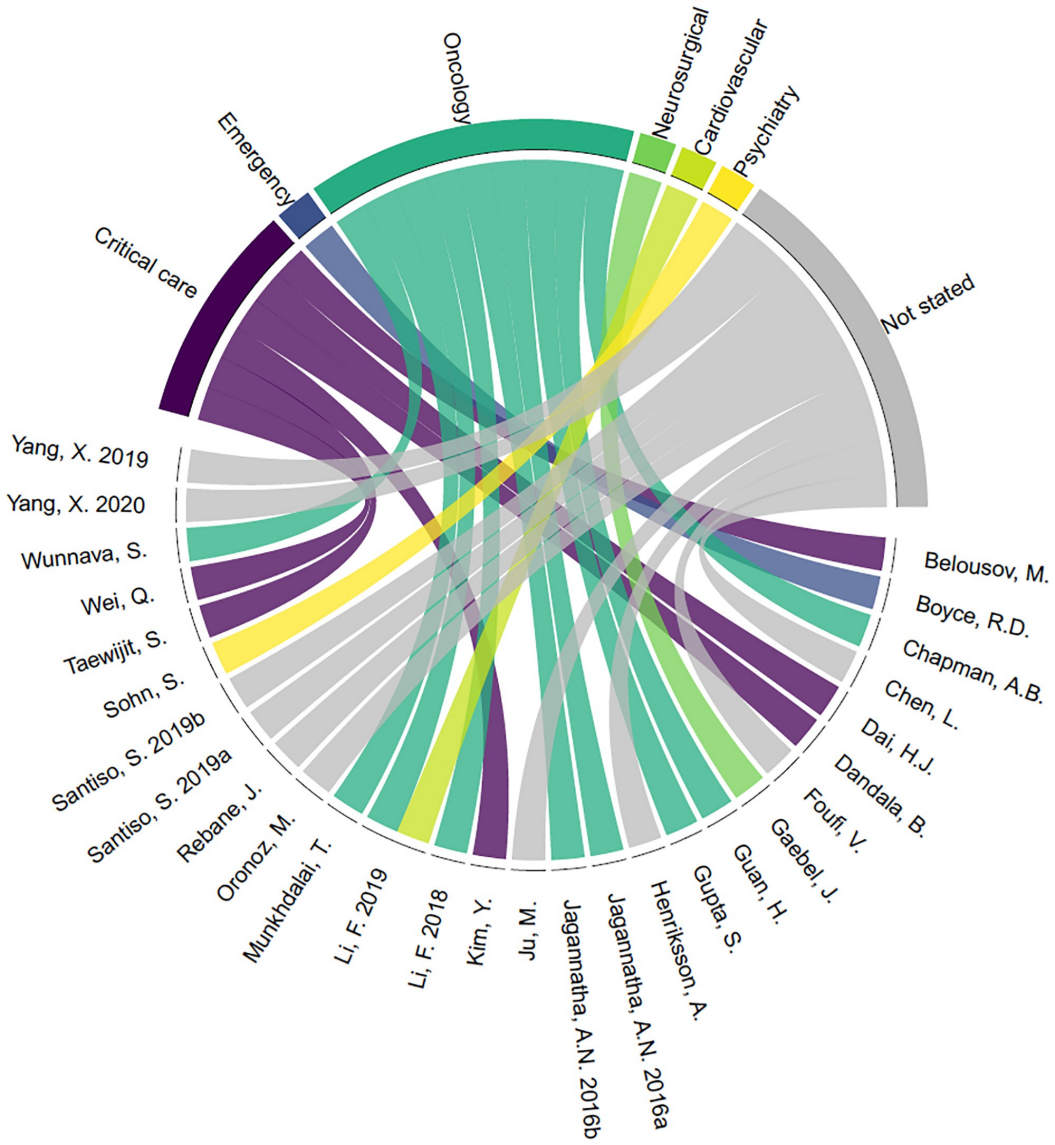
Patient type studied in each article (chord diagram from circlize [61]).

Just over one third of the studies (n = 10; 34.5%) did not state the clinical domain or patient type studied. Of the studies that did report this information, oncology (n = 9; 31%) and critical care (n = 6; 20.7%) were the most studied clinical domains (see Fig 4). The most commonly specified note type was a discharge summary or discharge letter (n = 10; 34.5%).

A total of seven (24.1%) of the studies wrote about participation in the 2018 n2c2 challenge [34, 43, 44, 48, 53, 56, 57] and four (13.8%) described participation in the MADE 1.0 challenge [49, 54, 56, 58] (see glossary). A further three studies (10.3%) did not participate in either challenge but used one or both of these challenge datasets [45, 50, 59]. Table 3 provides details on the datasets used in the studies.

### 3.6 Data preparation

#### 3.6.1 Annotation

**Fig 5:**
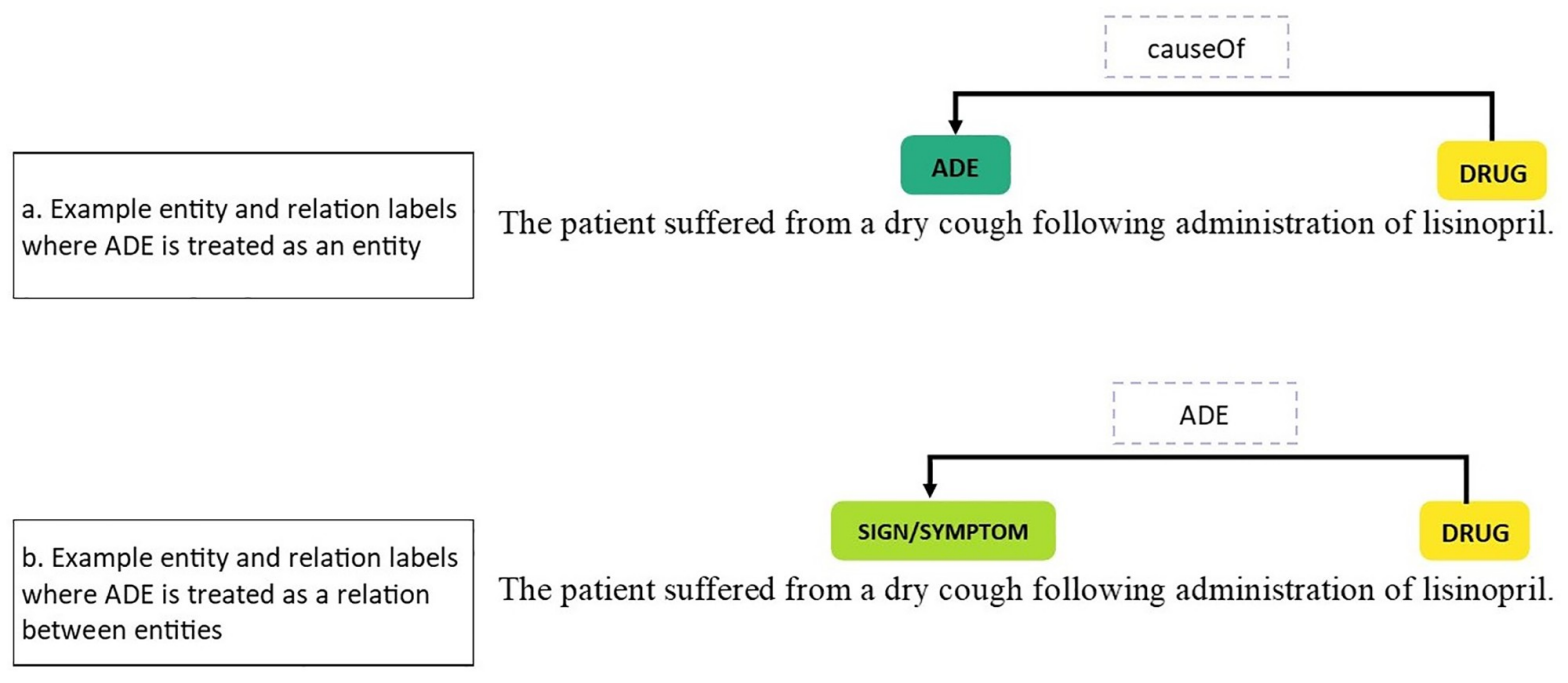
Definition of an ADE as an entity or as a relation in the text; solid coloured rectangles represent entities and dashed line boxes over linking arrows represent relationships between entities.

While some studies had access to labelled data (most notably those participating in the shared task challenges), ten studies (34.5%) reported annotating their own datasets. For the studies using entities, there were two approaches to defining ADEs. Some defined an ADE entity, and some defined an ADE as a relation between a drug entity and a non-drug entity (see Fig 5).

Two studies provided detailed accounts of creating a gold standard annotated corpus in a language other than English, and both made their annotation guidelines available. Oronoz et al. reported creation of a gold standard Spanish corpus of 75 documents, the creation of which took approximately one year [39]. Henriksson et al. did not state the length of time taken to create their corpus of 400 Swedish clinical narratives [33].

Five studies defined ADEs neither as entities nor relations, but at a higher level (sentence, document, or patient). Two studies annotated their data at the patient level, marking each patient as positive or negative for experiencing an ADE [36, 42]. For the studies with document-level classification, either documents or sentences were assigned binary labels indicating presence or absence of an ADE.

#### 3.6.2 Pre-processing

Most of the studies (n = 25; 86.2%) reported some form of typical NLP pre-processing tasks including sentence boundary detection, tokenization, and part-of-speech tagging. Some commented on difficulties encountered when applying off-the-shelf generic pre-processing tools to clinical text. Dandala et al. observed that sentence boundary detection and tokenization are difficult issues in clinical text as sentence ends are frequently denoted by newline characters rather than punctuation [45]. This was echoed in another paper where it was noted that several generic sentence segmentation tools did not perform well due to differences in punctuation patterns and the use of newline characters in formatting [43]. Four studies overcame this by building their own custom tokenizer or sentence splitter [36, 45, 48, 54].

#### 3.6.3 Other data preparation tasks

Four studies used tools for named entity normalization to match tokens in their data to a corresponding medical concept from a standardized ontology [31, 38, 41, 58]. These studies all had English language data. Tools used included cTAKES [62], NOBLE [63], MedEx [64], and MetaMap [65] and terminologies included SNOMED-CT (n = 2), ICD-9 (n = 1), MedDRA (n = 1), MeSH (n = 2), and RxNorm (n = 1).

Other data preparation tasks related to complexities in the data, such as class imbalance, duplicate sentences (due to copy-paste from previous notes), or overlapping entities. Class imbalance in particular was mentioned in several studies [33, 35, 39, 47, 51, 57, 59]. Santiso et al. reported an imbalance ratio of 1:222, where for each related drug-disease pair that is an adverse drug reaction, they had 222 such pairs that were not adverse drug reactions [51]. Class imbalance was tackled using a variety of methods including undersampling [42, 59], resampling [39], edge sampling [59], cost-sensitive learning [51], ensemble learning [51], and one-class classification [51].

### 3.7 Modelling

Tasks that were frequently described included named entity recognition (n = 17; 58.6%) and relation extraction and classification (n = 15; 51.7%). A diverse array of machine learning methods and models were reported in the studies. Several studies compared different methods or used ensembles of models in their tasks. Long Short Term Memory (LSTM) (n = 16; 55.2%) and Conditional Random Field (CRF) (n = 11; 37.9%) were the methods most frequently used. These methods are particularly suitable for NLP; LSTM because of its feedback connections which allow it to process sequences of information, and CRF because it does not assume that variables (in this case, words) are independent, and can therefore take context into account in making its predictions. Table 4 lists the methods.

**Table 4:** Methods, performance and evaluation measures.

Lead author	Method(s)	Model	Performance measure(s)	Evaluation measure(s)	How is over/underfitting and optimism in model performance handled/assessed?
**Belousov, M.** [57]	BiLSTM-CRF	RNN, Gr	P, R, F1	-	Hold-out cross validation, early stopping
**Boyce, R.D.** [31]	RF	DTE	P, R, F1, AUROC	-	Feature selection strategies, 10-fold cross validation
	CART	DT			
**Chapman, A.B.** [58]	CRF	Gr	F1	-	Hold-out validation
	RF	DTE			
**Chen, L.** [43]	BiLSTM	RNN	P, R, F1	-	Hyperparameter tuning (dropout, regulation, hidden unit size, learning rate)
**Dai, H.J.** [44]	Ensemble CRFs	Gr	P, R, F1	-	Hold-out development set, hyperparameter tuning
**Dandala, B.** [45]	BiLSTM-CRF	RNN, Gr	P, R, F1	Statistical significance (significance level 0.05)	-
**Foufi, V.** [40]	SVM, Naïve Bayes, Linear	PC	P, R, F1, accuracy	-	-
**Gaebel, J.** [32]	SVM	LC	P, R, F1	-	10-fold cross validation
**Guan, H.** [59]	BERT	TLM	P, R, F1, ΔF, ERR	McNamara test^a^	Parameter tuning
**Gupta, S.** [36]	BiLSTM	RNN	P, R, F1, AUROC	-	Parameter tuning
**Henriksson, A.** [33]	CRF	Gr	P, R, F1, accuracy	McNemar’s test	10-fold cross validation, hyperparameter tuning, L2 regularization
**Jagannatha, A.N. 2016a** [46]	BiLSTM, BiLSTM-CRF, BiLSTM-CRF with pairwise modelling	RNN, Gr	P, R, F1	Pairwise t-test for each fold in cross-validation	Cross validation, early stopping
**Jagannatha, A.N. 2016b** [47]	LSTM and GRU	RNN	P, R, F1	-	Early stopping, dropout, L2 regularization
**Ju, M.** [48]	BiLSTM	RNN	P, R, F1	-	-
**Kim, Y.** [34]	Ensemble of CRF, CRFext, BiLSTM, Searn	RNN, Gr	P, R, F1	Paired t-test	L1 and L2 regularization, 10-fold cross validation
**Li, F. 2018** [49]	BiLSTM-CRF with multitask learning	RNN, Gr	P, R, F1	-	Parameter regularization
**Li, F. 2019** [50]	MLP	ANN	P, R, F1	-	-
**Mitra, A.** [37]	BiLSTM-CRF	RNN, Gr	P, R, F1	-	Hyperparameter tuning, dropout, early stopping
**Munkhdalai, T.** [41]	SVM	LC	P, R, F1	-	Hyperparameter tuning (SVM), dropout (LSTM)
	LSTM, BiLSTM	RNN			
**Oronoz, M.** [39]	RF	DTE	P, R, F1	Mean and standard deviation on 500 replications	10-fold cross validation, parameter tuning
**Rebane, J.** [42]	RETAIN	RNN	F1, AUC	-	Dropout, regularization
**Santiso, S. 2019a** [51]	RF	DTE	TP, FN, FP, TN, P, R, F1, AUC	-	Bagging, boosting, stacking, weighting voting, majority voting
**Santiso, S. 2019b** [35]	Joint AB-LSTM	RNN	P, R, F1, AUC	Friedman test	L2 regularization, dropout
**Sohn, S.** [38]	Rules + decision tree	DT	P, R, F1	-	-
**Taewijit, S.** [52]	Multiple-instance learning with expectation maximization	G	P, R, F1	-	Parameter settings
**Wei, Q.** [53]	BiLSTM	RNN	P, R, F1	-	5-fold cross validation
	CRF	Gr			
	SVM	LC			
**Wunnava, S.** [54]	BiLSTM	RNN	P, R, F1	Pairwise t-test	Dropout, validation set (10%) to determine early stopping
**Yang, X. 2020** [56]	LSTM	Gr	P, R, F1	Statistical tests	Validation set, hyperparameter optimization (LSTM), 5-fold cross validation (SVM, RF, GB), grid search (SVM, RF, GB)
	SVM	LC			
	RF	DTE			
	GB	DT			
**Yang, X. 2019** [55]	LSTM-CRF	RNN, Gr	P, R, F1	-	Dropout (LSTM), Validation set (LSTM), 5-fold cross validation (SVM, RF), grid search (SVM, RF)
	SVM	LC			
	RF	DTE			

AB: attention-based bidirectional, BERT: Bidirectional Encoder Representations from Transformers, Bi: bidirectional, LSTM: long short term memory, CART: classification and regression tree, CRF: conditional random field, GB: gradient boosting, GRU: gated recurrent unit, MLP: multilayer perceptron, RETAIN: Reverse Time Attention, RF: random forest, SVM: support vector machine.

ANN: artificial neural network, DT: decision tree, DTE: decision tree ensemble, G: generative model, Gr: graphical model, LC: linear classifier, PC: probabilistic classifier, RNN: recurrent neural network, TLM: transformer-based language model.

P: precision, R: recall, F1: F1 score, ΔF: absolute F measure difference, AUC: area under the curve, AUROC: area under the receiver operator characteristic curve

^a^ Article states ‘McNamara test’ but related reference is for McNemar’s test.

### 3.8 Evaluation

#### 3.8.1 Performance evaluation

Just over half (n = 16; 55.2%) of the articles reported the rationale for choice of performance metric, with metric used in challenge (n = 11; 37.9%) and standard or commonly used metric (n = 4; 13.8%) given as the main reasons. One article chose their metric given the unequal distribution of classes in the dataset, as the metric allowed them to highlight the positive class [51]. In total, nine (31%) articles reported an evaluation metric. Performance and evaluation methods for each article are described in Table 4.

The articles reported either precision and recall, or F1 score, or both. Some reported micro-averages of precision, recall, and F1 score which are useful when a system is applied to a multi-class classification problem, and gives an impression of the performance on individual classes (for example, ADE entity or ADE relation). Eight articles (27.6%) reported these performance measures under either strict or lenient matching or both; the other articles did not state whether strict or lenient matching was applied.

All articles reported the overall performance of their models across all prediction classes, whether it was entities (for example Diagnosis, ADE, Drug, Strength, Dose), relations (Drug-Dose, Drug-Symptom, Drug-Disease), or a combination of both (end-to-end performance). Additionally, some articles reported the performance on predicting just the ADE entity or ADE relation class; in all cases this was lower than the performance across all entities or relations (see Table 5).

**Table 5:** Summary of single best reported result for overall performance and ADE entity or relation class performance by task.

Task	Best performing method	Performance metric	Best overall result	Best ADE entity/ relation result
**NER**	Ensemble CRF-BiLSTM-CRF-Joint [53]	Lenient F1 score	0.9345	0.5295
	Positional-Joint BiLSTM-CRF [45]	Lenient F1 score	0.934	0.518
	RCNN-KB [56]	Lenient F1 score	0.9292	-
	NN [48]	Lenient micro-F1 score	0.9278	-
	Stacked ensemble CRF-Searn-RNN [34]	Lenient micro-averaged F1 score	0.9266	0.2711
	CNN-BiLSTM-CRF [44]	Lenient F-score	0.913	0.3875
	BiLSTM-CRF [57]	Lenient micro-averaged F1 score	0.9123	0.405
	SVM [41]	F1 score	0.891	0.85
	Skip-chain RNN-CRF [46]	Relaxed micro-averaged F-score	0.8632	-
	BiLSTM-CRF [43]	Lenient micro-averaged F1 score	0.8497	0.4329
	HardMTL [49]	Micro-averaged F1 score	0.845	0.455
	CRF [33]	Micro-averaged F1 score	0.835	-
	DLADE (BiLSTM-CRF) [54]	Micro-averaged F1 score	0.829	-
	MADEx (BiLSTM-CRF) [55]	F1 score	0.8233	-
	CRF [58]	Micro-averaged F1 score	0.809	0.511
	GRU [47]	Micro-averaged F-score	0.8031	-
	LM-BiLSTM-CRF with BioBERT/RoBERTa [37]	Micro-averaged F1 score	0.76	-
**RE**	RF [51]	Micro-averaged F1 score	0.998	-
	SVM [56]	Lenient F1 score	0.9635	-
	Joint + rule-based post-processing [53]	Lenient F1 score	0.963	0.8502
	Transductive learning approach [52]	F1 score	0.954	-
	Att-BiLSTM [43]	Lenient micro-averaged F1 score	0.9442	-
	Joint AB-LSTM [35]	Micro-averaged F1 score	0.938	-
	SVM [34]	Micro-averaged F1 score	0.9359	-
	Positional-Joint BiLSTM-CRF [45]	Lenient F1 score	0.894	0.46
	RF [58]	Micro-averaged F1 score	0.881	-
	MLP [50]	F1 score	0.872	-
	BERT + Edge sampling [59]	F-measure	0.83	-
	Rules + decision tree [38]	F score	0.745	-
	HardMTL [49]	Micro-averaged F1 score	0.667	-
	RF [39]	F1 score	0.426	-
	RF [33]	Macro-averaged F1 score	0.343	0.202
**End-to-end/integrated NER-RE task**	CNN-RNN + rule-based post-processing [53]	Lenient F1 score	0.8905	0.4755
	LSTM-CRF+GB [56]	Lenient F1 score	0.888	-
	BiLSTM-CRF-Att-BiLSTM [43]	Lenient micro-averaged F1 score	0.7938	0.3303
	CRF-RF [58]	Micro-averaged F1 score	0.612	-
**Patient labelling**	RETAIN-TERF [42]	Micro-averaged F1 score	0.83	-
	CNN [36]	F1 score	0.752	-
**Document labelling**	Linear classifier [40]	Accuracy	0.94	-
	CART [31]	F-measure	0.74	-
**Sentence annotation**	SVM [32]	F-measure	0.577	-

AB: attention-based bidirectional, BERT: Bidirectional Encoder Representations from Transformers; Bi: bidirectional, CART: classification and regression tree, CNN: convolutional neural network, CRF: conditional random field, DLADE: dual-level embedding for adverse drug event detection, GB: gradient boosting, GRU: gated recurrent unit, KB: knowledge embedding; LM: language modelling, LSTM: long short term memory, MLP: multilayer perceptron, MTL: multi-task learning, NER: named entitiy recognition, RETAIN-TERF: an interpretable RNN model with Text features and Early Retain Fusion, RE: relation extraction, RF: random forest, RNN: recurrent neural network, SVM: support vector machine.

Many methods were employed to account for model complexity such as hyperparameter tuning and regularization, and for measuring unbiased model performance, including k-fold cross validation. None of the articles indicated a cut-off value for determining a good performance in advance of performing the analysis.

#### 3.8.2 Validation strategy

Of the articles reporting performing internal validation on their models, seven (24.1%) reported k-fold cross validation [32, 35, 36, 46, 47, 52] and 11 (37.9%) reported using a holdout validation set [39, 42–44, 48, 49, 51, 54, 57–59]. The need for external validation was mentioned by five articles (17.2%) [31, 41, 45, 49, 58].

#### 3.8.3 Error analysis

In total 12 articles (41.4%) report an analysis of errors made by their models. Table 6 provides examples of errors reported by at least two articles. Of those who gave details of an error analysis, five discussed possible changes to their methods on the basis of this analysis.

**Table 6:** Common errors described in error analyses.

Error	Error description and example	References
**Intersentential relations missed**	Relation between drug and related entities missed due to entities in different sentences or long distance between entities in the text	[33, 34, 43, 45, 49, 50]
	*“Haldol and* ***Tradazone*** *have been attempted at rehab without good effect and were discontinued due the drowsiness as well as (per ED report) some symptoms of lip smacking that were thought to be* ***tardive dyskinesia***.*”*–relation between tradazone and tardive dyskinesia missed [43]	
**Entity confusion by model or annotator**	Similar entities mislabelled as each other, such as dosage and strength, route and form, ADE and indication/reason, ADE and sign/symptom	[34, 45, 49, 57, 58]
	*“She received* ***one litre*** *of normal saline”*–annotators had difficulty determining if “one litre” is a Dose or a Strength entity [57]	
**Omission in annotation**	Model predicts entity that is not annotated in corpus, or entity annotated in one instance but not in another instance	[43, 57, 58]
	“*Gabapentin 300 mg* ***3 times daily***”–Frequency was missed during annotation [58]	
**Failure to take account of attributes**	One of the entities is negated, speculative, or resolved but the relation is still identified	[36, 37, 45]
	“*no source of bleeding*”–‘bleeding’ annotated as bleeding event [37]	
**Inconsistent annotation**	Entity span boundaries differed within the corpus for the same entity	[37, 57]
	In one note “***acute bleeding***” annotated, but in another note only the word “***bleeding***” annotated [37]	
**Entity missed**	Entity not identified due to misspelling or abbreviation	[34, 45]
	*“…acute kidney injury due to* ***genta****”*–drug entity missed due to use of abbreviation “genta” for gentamicin	
**Multiple entity labels apply to the same entity**	Multiple labels can apply to an entity depending on the context of its related entities	[45, 58]
	*“She was on* ***furosemide*** *and became* ***hypotensive*** *requiring* ***norepinephrine****”*–“hypotensive” is an Indication for norepinephrine but an ADE for furosemide [45]	

### 3.9 Deployment

None of the articles described implementation of their NLP application in clinical practice, but two did mention plans for implementation as part of future work [31, 41]. To our knowledge, neither has published follow-up articles detailing a deployed system.

## 4. Discussion

### 4.1 Main findings

We identified 29 studies that matched our inclusion criteria that reported on the application of NLP for the detection of ADEs. Our scoping review shows that at present the limiting step in creating NLP-based systems for ADE detection in hospitalized patients is data preparation including annotation and pre-processing of text. This seems especially problematic for languages other than English. Also, although many off-the-shelf tools exists for data pre-processing, their usefulness for pre-processing clinical text is limited. These findings may explain the limited evidence of externally validated models or implementation of NLP applications in clinical practice. Although the included studies encompass diverse clinical domains, setting, narratives and methods used, LSTM and CRF (or a combination of these) methods are most frequently used in ADE detection from clinical narratives.

### 4.2 Business understanding

Just under one third of the studies explicitly reported clinical involvement. This involvement was limited to annotation of notes, annotation scheme design, and clinical chart review. None of the studies reported clinical involvement in areas such as overall design or interpretation of the results, although it is possible that this is a gap in reporting. These findings are similar to a recent review on clinical involvement in the development of machine learning clinical decision support systems, in which 21% of the studies on component development involved clinical experts in their process [66]. Simon et al. have strongly recommended that collaboration between technical and clinical teams is not only important but should be clinician-led when developing artificial intelligence solutions for medicine, as the two perspectives may not always agree [67].

### 4.3 Data preparation

Many of the difficulties encountered by the studies occurred at the data preparation stage when annotating and pre-processing the data. Off-the-shelf pre-processing tools perform poorly on clinical text for several reasons. The language is domain-specific, abbreviations and jargon are frequently used, words which can be inferred from context are skipped, and whitespace and new lines rather than punctuation are used in formatting [68]. This differs from the text used to train the standard pre-processing tools, which generally follow accepted grammar and punctuation rules. Some studies tackled this problem by building their own custom pre-processing tools. Other pre-processing tasks such as named entity normalization were only performed in studies where the language of the data is English. The majority of the tools available for this task are for English; therefore, researchers seeking to match their clinical narratives to standard terminologies in other languages face the additional barrier of building own tools from scratch. Joining efforts on national level in creating custom tools for clinical narratives in a specific language could partly circumvent this barrier [69]. Adapting tools that work well for English to another language could be another promising path [70]. Studies in which pre-processing tools for clinical narratives are compared are needed to support researchers in making a choice between the growing number of such tools [12, 71].

The few studies that described in detail the creation of an annotated gold standard corpus describe a large effort to create relatively small datasets [33, 39]. A 2020 review of studies on clinical NLP similarly noted that annotation is a bottleneck step in the use of clinical text data [60]. The effort required could be reduced by employing semi-automated methods to augment the annotation process. Such methods have demonstrated relatively small time savings of 13.85% to 21.5% per entity by employing dictionary-based pre-annotations, which were then checked (and corrected if necessary) by a human annotator [72]. Another study found that pre-annotations reduced the number of hand annotations necessary by 28.9% with consequent lower annotation time and higher inter-annotator agreement [73]. The availability of labelled data from the shared-task challenges greatly enhanced the efforts in applying NLP for ADE detection. Luo et al. [9] anticipated the significant impact of shared-task challenges in promoting and accelerating efforts in this area, the datasets from which were the basis for many of the articles included in this review. Just like for pre-processing tools, joining forces in creating annotated gold standard corpora for a specific task via shared-task challenges especially for languages other than English, should be encouraged.

The error analyses reported by the studies point to the importance and difficulty of the annotation task. Choices in annotation scheme design and accuracy of annotation scheme application both contributed to errors found in the studies, while other errors arose where annotators could not easily identify the correct clinical entity label to apply. These issues can be partly tackled by treating an ADE as a relation between a drug and non-drug entity and by making annotation schemes detailed and explicit. In particular, the representation of an ADE as an entity rather than as the relationship between a drug and non-drug entity led to avoidable errors. Where ADE is treated as an entity, the same symptom can be an adverse event in the context of one drug, but an indication in the context of another drug [45]; this makes it more difficult to accurately identify the ADE entity. For this reason we suggest that ADE should be treated as a relation between a drug and symptom entity, and not as an entity in itself. Treating an ADE as a relation between a drug and symptom entity reflects how clinicians think about such patterns in clinical data.

In their review of NLP in incident reporting and adverse event analysis, Young et al. noted that manual annotation is treated as a gold standard, yet the accuracy of the annotations determines the validity of the model accuracy measurements [74]. We agree that annotation accuracy plays an important role and also that annotation scheme design and choice of entity labels contributes to the validity of the results. The issues described in the studies could be tackled by involving clinicians in the process of designing and implementing an annotation scheme. Clinical narratives are designed to be interpreted by clinical readers, and clinicians have the requisite knowledge to interpret clinical text so as to derive maximum meaning from the annotated corpora. Clinicians also have the medical knowledge to apply these schemes correctly or to train lay annotators in their correct application. Clinicians can act as an interpreter between the written narrative and the process of automated extraction of data to ensure information is extracted as accurately as possible.

### 4.4 Modelling and evaluation

The included studies describe a variety of methods for the NER and RE tasks, and although variations of LSTM and CRF were most frequently seen, making a fair comparison between the reported methods is difficult. Factors such as dataset size, annotation quality and degree of model tuning affect performance of the methods. Additionally, variations on performance metric calculation and reporting make it difficult to compare between publications; although most papers reported the F1 score, the types of F1 score (micro- or macro-averaged) and the conditions under which it was reported (strict or lenient matching) varied, if they were reported at all. Indeed, even where the same type of metric appears to be reported, it may not have been calculated in the same way; a recent technical note highlighted that there are two different methods to calculate the macro-averaged F1 score which can differ in outcome by as much as 0.5 [75]. Both methods have each been described in a widely cited paper [76, 77] and the choice of formula is seldom reported when using the macro-averaged F1 score. In addition, some studies did not focus on optimizing performance of their model, but on other factors relevant to ADE detection such as correcting for class imbalance, annotation scheme design, or corpus creation. The performance of the model also depends on the task, as a model may be well suited to RE but be less suited to NER. Comparison of optimized model performances for the same task, on the same dataset, and using the same evaluation script (such as occurs with the shared task challenges) is the most fair way to evaluate which models are suitable for the particular task of ADE detection in clinical narratives [23, 24].

Overall performance of the systems was generally high but a steep drop in performance was reported when focusing on only the ADE entity or ADE relation class. This is because non-ADE entities such as drug names are relatively consistent in the data (“furosemide” will always refer to a drug name in the text) but this is not the case for ADEs, as “cough” can be an ADE in the context of lisinopril, but an indication in the context of codeine, or a symptom in the context of tuberculosis. This makes an ADE more complex to identify. Given that we are interested in detecting ADEs, the ability of systems to detect these ADE mentions in the text is more important than overall performance. When assessing performance it is therefore important to take into account performance on the ADE class and not just overall performance, as overall performance gives an artificially inflated impression of the ability to identify of ADEs in the text. This also reinforces our assertion that an ADE should be represented in data annotation as a relation between a drug and non-drug entities, to allow for accurate and consistent labelling of the data.

It is worth mentioning that in many of the studies where ADE is treated as an entity, the number of ADE entities can be over 1,000 [34, 43, 44, 56–58, 78, 79], and is usually higher than the number of notes included in the dataset. For example, the 2018 n2c2 challenge dataset used by seven studies [34, 43, 44, 48, 53, 56, 57] consists of 505 annotated documents. These 505 annotated documents contain 1,584 ADE entities (according to the challenge organizers [23]). At first glance this may seem like an extraordinarily high number of ADEs since the prevalence of ADEs varies between 1.9 to 57.9 ADEs per 100 patients [2]. However, as studies included in this review focus on NLP methods, several carefully preselected their datasets so that the notes would be more likely to contain ADE mentions [31, 33, 37, 40]; Henry et al. in describing the preparation of the n2c2 dataset ensured that each of the 505 discharge summaries include at least one ADE mention [23]. Therefore the number of ADE entities reported in the datasets does not necessarily reflect the number of ADEs in the studied patient populations. Additionally, none of the studies included in our review conducted a formal causality assessment between drug and adverse events found in the notes such as Naranjo probability scale or the World Health Organization Collaborating Center for International Dug Monitoring, the Uppsala Monitoring Center (WHO-UMC) criteria [80, 81]. Also, in the study by Boyce et al. [31] as illustrated in Fig 1 notes were labeled as containing ADE mentions in cases bleeding and a drug known to cause bleeding were both mentioned in the same note, yet without the relation mentioned between them by the clinician. Both such practice and lack of formal causality assessment may explain the inflated ADE numbers. We strongly advise to use best practices for assessing whether or not ADEs are present in the clinical notes, in order to create a corpus to learn on. At present, most used best practice is a manual chart review by medical experts using formal causality assessment criteria [80]. Furthermore, although there are 1,584 ADE entities in the n2c2 dataset, the same dataset contains over 26,000 drug entities and over 80,000 entities overall, making the ADE entities a small portion of the total [23], which is similar in other datasets across the included studies. This disparity between the total number of entities/relations and ADE entities/relations made class imbalance a consideration in many of the studies [33, 35, 39, 47, 51, 57, 59].

While the proportion of papers reporting internal validation steps was high, only one paper reported external validation. Similar to the lack of external validation seen here, Spasic and Nenadic noted no hard evidence of generalizability or transferability in the studies included in their clinical NLP review [60]. Opportunities for external validation may be limited by the availability of data. Luo et al. in their 2017 paper noted that almost all of their studies focus on EHRs limited to within their own institutions [9]. We noted a shift in this trend, with a close to 50/50 split between the use of data from own institutions and from publicly available datasets. Shared tasks challenges such as n2c2 and MADE 1.0 in 2018 have increased the availability of labelled data, which are invaluable resources in this domain that can be used for external validation of other English language datasets.

### 4.5 Deployment

None of the included articles discussed the practical application of NLP models for ADE detection in the clinical setting. Boyce et al. state that their model could be deployed as a trigger tool to detect drug-related bleeding mentions in Emergency Department notes, after it had been externally validated [31]. To carry out any such implementation one would require not just buy-in from the EHR vendors, but also clinical involvement in the design and implementation of such a system. This is essential to ensure that any such systems aligns with the needs and workflows of clinicians and therefore is taken up and not a wasted investment. Researchers in the United Kingdom have demonstrated the utility of a deployed NLP system both in identifying patients for clinical trial participation and for converting clinical narratives to structured data in real-time (thereby removing the need for double data entry) [82]; more such projects are needed to generate interest and enthusiasm for the implementation of NLP systems to exploit the rich data hidden in clinical narratives.

### 4.6 Strengths and limitations

We created a framework for NLP workflow (Fig 1) based on CRISP-DM for this review and this framework can be understood as a supervised machine learning pipeline for NLP. The sequential steps of the pipeline can be applied to detect ADEs in clinical narratives.

We identified strengths and limitations of current research, and promising direction for future studies. Furthermore, both quantitative and descriptive data, including details of error analysis, are reported. This knowledge is crucial for data scientists to optimize performance of NLP pipelines for the task of ADE detection. It also helps clinicians and pharmacists to understand the value of NLP for their practice and how they could contribute to the development of more robust and clinically valuable NLP pipelines for ADE detection. In addition to standard literature databases MEDLINE and EMBASE, the pre-print server, arXiv, is included in our search strategy to look for state-of-the-art technical literature.

A limitation of this review is that we critically appraised the methods in the papers based on our own choice of tools. However, currently no validated assessment tool or reporting guideline specific to publications on clinical NLP exists. The upcoming artificial intelligence extension to the Transparent Reporting of a multivariable prediction model of Individual Prognosis Or Diagnosis (TRIPOD) statement and PROBAST tool (TRIPOD-AI and PROBAST-AI) [83] may provide more clarity on this issue, but at this time there is a gap for assessing clinical NLP models.

### 4.7 Future directions

Future work should investigate semi-automated methods to reduce the manual effort required to create annotated corpora to train NLP models, and examine how NLP can be deployed to detect ADEs in clinical practice. Making annotated corpora available for others to use will facilitate the training of data-hungry deep learning models and enable external validation. Furthermore, adding clinicians and pharmacists as team members when applying NLP for ADE detection should be a standard practice, since their expertise is needed to ensure high quality annotated data is created, and to elicit best suited strategies to implement NLP models into clinical practice. In order to assess the value of the future NLP pipelines, the reporting pratices must align with available and future reporting standards. Lastly, the recent advances in weakly supervised machine learning methods present new and exciting opportunities worth exploring to support NLP application for ADE detection, for example, helping in the annotation task [84].

### 4.8 Conclusions

The studies included in the review demonstrate that it is feasible to extract information on ADEs from clinical narratives using NLP. This is especially useful given that these data has the potential to be reused for multiple purposes, including routine ADE monitoring, clinical decision support, and research. Clinical involvement appears low and there is potential for clinicians to play an active role in the design and implementation of these types of systems. Performance on the ADE entity or ADE relation class is low compared to overall performance. The studies examined here demonstrate that multiple modelling approaches are available, but more work is needed in data preparation and deployment stages of the NLP process. Especially for languages other than English, extra barriers are present like lack of ontology-matching tools. When annotating corpora, treating an ADE as a relation between a drug and non-drug entity seems the best practice. Although the included studies encompass diverse clinical domains, setting, narratives and methods used, LSTM and CRF (or a combination of these) methods are most frequently used in ADE detection from clinical narratives.

## Supporting information

S1 Checklist PRISMA ScR fillable. (PDF)

S1 File Search queries. (DOCX)
