# Supplementary material for: Adverse drug event detection using natural language processing: A scoping review of supervised learning methods
Source: PLoS One. 2023 Jan 3;18(1):e0279842. doi: 10.1371/journal.pone.0279842 (PMC9810201; doi:10.1371/journal.pone.0279842)
Supplement: S1 File — (DOCX) [file pone.0279842.s002.docx]

## Article information

### Article title

Adverse drug event detection using natural language processing: a scoping review of supervised learning methods

### Author information

Rachel M. Murphy^a, b^, Joanna E. Klopotowska ^a, b^, Nicolette F. de Keizer ^a, b^, Kitty J. Jager ^a, b^, Jan Hendrik Leopold ^a, b^, Dave A. Dongelmans ^b, c^, Ameen Abu-Hanna ^a, b^, Martijn C. Schut^a, b^

^a^ Amsterdam UMC (location AMC), Department of Medical Informatics, Amsterdam, the Netherlands

^b^ Amsterdam Public Health Research Institute, Amsterdam, The Netherlands

^c^ Amsterdam UMC (location AMC), Department of Intensive Care Medicine, Amsterdam, the Netherlands

## Search strategies

### Medline

*Concept: ADE*

1. (adverse adj3 drug adj3 reaction?).ti,ab,kf
2. (adverse adj3 drug adj3 event?).ti,ab,kf
3. ADE?.ti,ab,kf
4. ADR?.ti,ab,kf
5. side adj1 effect?.ti,ab,kf
6. drug adj1 induced.ti,ab,kf
7. drug adj1 related.ti,ab,kf
8. exp Adverse Drug Reaction Reporting Systems/ [MeSH term]
9. drug-adverse adj1 event.ti,ab,kf
10. 1 or 2 or 3 or 4 or 5 or 6 or 7 or 8 or 9 [ADE group]

*Concept: Clinical notes*

1. note?.ti,ab,kf
2. text?.ti,ab,kf
3. summar*.ti,ab,kf
4. exp Patient Discharge Summaries/ [MeSH term]
5. letter?.ti,ab,kf
6. narrative?.ti,ab,kf
7. unstructured.ti,ab,kf
8. report?.ti,ab,kf
9. 11 or 12 or 13 or 14 or 15 or 16 or 17 or 18 [NOTE group]

*Concept: natural language processing*

1. exp Natural Language Processing/ [MeSH term]
2. (natural adj1 language adj1 processing).ti,ab,kf
3. (medical adj1 language adj1 processing).ti,ab,kf
4. nlp.ti,ab,kf
5. (text adj1 mining).ti,ab,kf
6. exp Data Mining/ [MeSH term]
7. (data adj1 mining).ti,ab,kf
8. embedding?.ti,ab,kf
9. (named adj1 entity adj1 recognition).ti,ab,kf
10. 20 or 21 or 22 or 23 or 24 or 25 or 26 or 27 or 28 [NLP group]

*Concept: social media*

1. exp Social Media/ [MeSH term]
2. (social adj1 media).ti,ab,kf
3. tweet*.ti,ab,kf
4. twitter.ti,ab,kf
5. 30 or 31 or 32 or 33 [SOCIAL MEDIA group]

*Combined concept groups*

1. 10 and 19 and 29 [three main concepts]
2. 35 not 34 [three main concepts not social media]

### EMBASE

*Concept: ADE*

1. (adverse adj3 drug adj3 reaction?).ti,ab,kw
2. (adverse adj3 drug adj3 event?).ti,ab,kw
3. ADE?.ti,ab,kw
4. ADR?.ti,ab,kw
5. exp side effect/
6. side adj1 effect?.ti,ab,kw
7. drug adj1 induced.ti,ab,kw
8. drug adj1 related.ti,ab,kw
9. drug-adverse adj1 event.ti,ab,kw
10. exp adverse drug reaction/
11. 1 or 2 or 3 or 4 or 5 or 6 or 7 or 8 or 9 or 10 [ADE group]

*Concept: Clinical notes*

1. note?.ti,ab,kw
2. text?.ti,ab,kw
3. summar*.ti,ab,kw
4. letter?.ti,ab,kw
5. narrative?.ti,ab,kw
6. unstructured.ti,ab,kw
7. report?.ti,ab,kw
8. (discharge adj1 summar*).ti,ab,kw
9. 12 or 13 or 14 or 15 or 16 or 17 or 18 or 19 [NOTE group]

*Concept: natural language processing*

1. exp natural language processing/
2. (natural adj1 language adj1 processing).ti,ab,kw
3. (medical adj1 language adj1 processing).ti,ab,kw
4. nlp.ti,ab,kw
5. (text adj1 mining).ti,ab,kw
6. exp data mining/
7. (data adj1 mining).ti,ab,kw
8. embedding?.ti,ab,kw
9. (named adj1 entity adj1 recognition).ti,ab,kw
10. 21 or 22 or 23 or 24 or 25 or 26 or 27 or 28 or 29 [NLP group]

*Concept: social media*

1. (social adj1 media).ti,ab,kw
2. exp social media/
3. twitter.ti,ab,kw
4. tweet*.ti,ab,kw
5. 31 or 32 or 33 or 34 [SOCIAL MEDIA group]

*Combined concept groups*

1. 11 and 20 and 30 [three key concepts]
2. 36 not 35 [three key concepts not social media]

### arXiv

Search 1:

note* OR text IN Abstract

AND drug* OR medication* IN Abstract

Search 2:

named entity recognition OR embedding IN Abstract

AND drug* OR medication* IN Abstract
